# Distinct Roles of Honeybee Gut Bacteria on Host Metabolism and Neurological Processes

**DOI:** 10.1128/spectrum.02438-21

**Published:** 2022-03-10

**Authors:** Zijing Zhang, Xiaohuan Mu, Yao Shi, Hao Zheng

**Affiliations:** a College of Food Science and Nutritional Engineering, China Agricultural University, Beijing, China; Nanchang University

**Keywords:** *Apis mellifera*, microbiome, metabolism, behavior, gut-brain axis

## Abstract

The honeybee possesses a limited number of bacterial phylotypes that play essential roles in host metabolism, hormonal signaling, and feeding behavior. However, the contribution of individual gut members in shaping honeybee brain profiles remains unclear. By generating gnotobiotic bees which were mono-colonized by a single gut bacterium, we revealed that different species regulated specific modules of metabolites in the hemolymph. Circulating metabolites involved in carbohydrate and glycerophospholipid metabolism pathways were mostly regulated by *Gilliamella*, while *Lactobacillus* Firm4 and Firm5 mainly altered amino acid metabolism pathways. We then analyzed the brain transcriptomes of bees mono-colonized with these three bacteria. These showed distinctive gene expression profiles, and genes related to olfactory functions and labor division were upregulated by *Lactobacillus*. Interestingly, differentially spliced genes in the brains of gnotobiotic bees largely overlapped with those of bees unresponsive to social stimuli. The differentially spliced genes were enriched in pathways involved in neural development and synaptic transmission. We showed that gut bacteria altered neurotransmitter levels in the brain. In particular, dopamine and serotonin, which show inhibitory effects on the sensory sensitivity of bees, were downregulated in bacteria-colonized bees. The proboscis extension response showed that a normal gut microbiota is essential for the taste-related behavior of honeybees, suggesting the contribution of potential interactions among different gut species to the host’s physiology. Our findings provide fundamental insights into the diverse functions of gut bacteria which likely contribute to honeybee neurological processes.

**IMPORTANCE** The honeybee possesses a simple and host-restricted gut community that contributes to the metabolic health of its host, while the effects of bacterial symbionts on host neural functions remain elusive. We found that the colonization of specific bee gut bacteria regulates distinct circulating metabolites enriched in carbohydrate, amino acid, and glycerophospholipid metabolic pathways. The brains of bees colonized with different gut members display distinct transcriptomic profiles of genes crucial for bee behaviors and division of labor. Alternative splicing of genes related to disordered bee behaviors is also mediated. The presence of gut bacteria promotes sucrose sensitivity with major neurotransmitters being regulated in the brain. Our findings demonstrate how individual bee gut species affect host behaviors, highlighting the gut-brain connections important for honeybee neurobiological and physiological states.

## INTRODUCTION

The honeybee has been widely used as a model animal for perception, cognition, and social behavior studies (1). As eusocial insects, honeybees have distinct behavioral structures characterized by a complex range of interactive behaviors within the hive. These sophisticated behaviors rely heavily on sensory sensitivity. For example, it is critical for foraging bees to perceive visual, olfactory, gustatory, and mechanosensory cues at feeding sites (pollen and nectar sources). Bees can also use social information to recognize nestmates and avoid potential predators, behaviors which also depend on sensory systems (2). To study the neurobiology and behavioral physiology of honeybees, a set of established methods is available to quantify their sophisticated behaviors, such as sensory responsiveness, associative appetitive learning and memory, and hive behavioral observation (3). It has been documented that nutrients such as carbohydrates, amino acids, and lipids are required for normal behaviors (4). Cognitive performance can be impaired due to polyunsaturated fatty acid deficiency (5). Moreover, behavioral shifts are associated with changes in brain gene expression levels in honeybees (6, 7). Neurotransmitters, such as biogenic amines, have an arousing effect on sensitivity to sensory inputs, learning performance, and foraging behavior (8).

The bidirectional microbiota-gut-brain axis influences various complex aspects of behavior across the animal kingdom. The gut microbiota can modulate homeostasis and behavior in its animal host through chemical communication with the nervous system. Previous studies have suggested that the honeybee gut microbiota contributes to host brain physiology and behavior phenotypes. Specifically, the gut microbiota can alter endocrine signaling and thus sucrose sensitivity in honeybees (9), as well as nestmate recognition involving cuticular hydrocarbon profiles (10). The levels of biogenic amines (serotonin, dopamine, octopamine) implicated in bee behaviors are lower in newly emerged bees, which have an immature gut community (11).

Like mammals, honeybees harbor a highly specialized but simple gut microbiota that has evolved specific interactions with the host. The gut community of honeybees is dominated by 8 to 10 bacterial phylotypes comprising over 97% of the community (12–14). *Gilliamella*, *Snodgrassella*, *Bifidobacterium*, *Lactobacillus* Firm4 and Firm5, and *Bartonella* are the major bacterial phylotypes, and can be cultivated in the laboratory. Recently, the causal roles of honeybee gut bacteria in host nutrition, weight gain, and endocrine signaling have been extensively studied (15), and these studies benefit from the availability of microbiota-free (MF) bees (9, 16). In particular, *Gilliamella*, *Bifidobacterium*, and *Lactobacillus* Firm5 can degrade diet polysaccharides in the honeybee gut, which might assist pollen perforation, resulting in the release of nutrient-rich content (17–19). In addition, interspecies interactions facilitate carbohydrate metabolism and amino acid synthesis, thus benefiting the host (18, 20). Untargeted metabolomics have revealed that a plethora of organic acids accumulate in the presence of gut bacteria, which may have pivotal functional consequences for the host’s physiology (16, 18). Indeed, the gut microbiota affects the host metabolism in both the gut and the hemolymph (9, 16). It has been shown that the honeybees with a conventional (CV) gut microbiota are more sensitive to sugars and possessed increased levels of insulin-related genes (9). However, a detailed understanding of how specific honeybee gut members contribute to host behavior and neurological processes is needed.

Here, we established gnotobiotic bees mono-colonized with different gut bacteria to disentangle the distinct roles of honeybee gut members on the host’s metabolism and neurological functions. Multiomics analysis revealed that gut bacteria impact circulating metabolic profiles and transcriptional programs in the brain. Our results showed that the presence of specific gut bacteria was sufficient to alter neurotransmitter concentrations and promote host perception and cognition.

## RESULTS

### Gut bacteria alters circulating metabolomic profiles.

**FIG 1:**
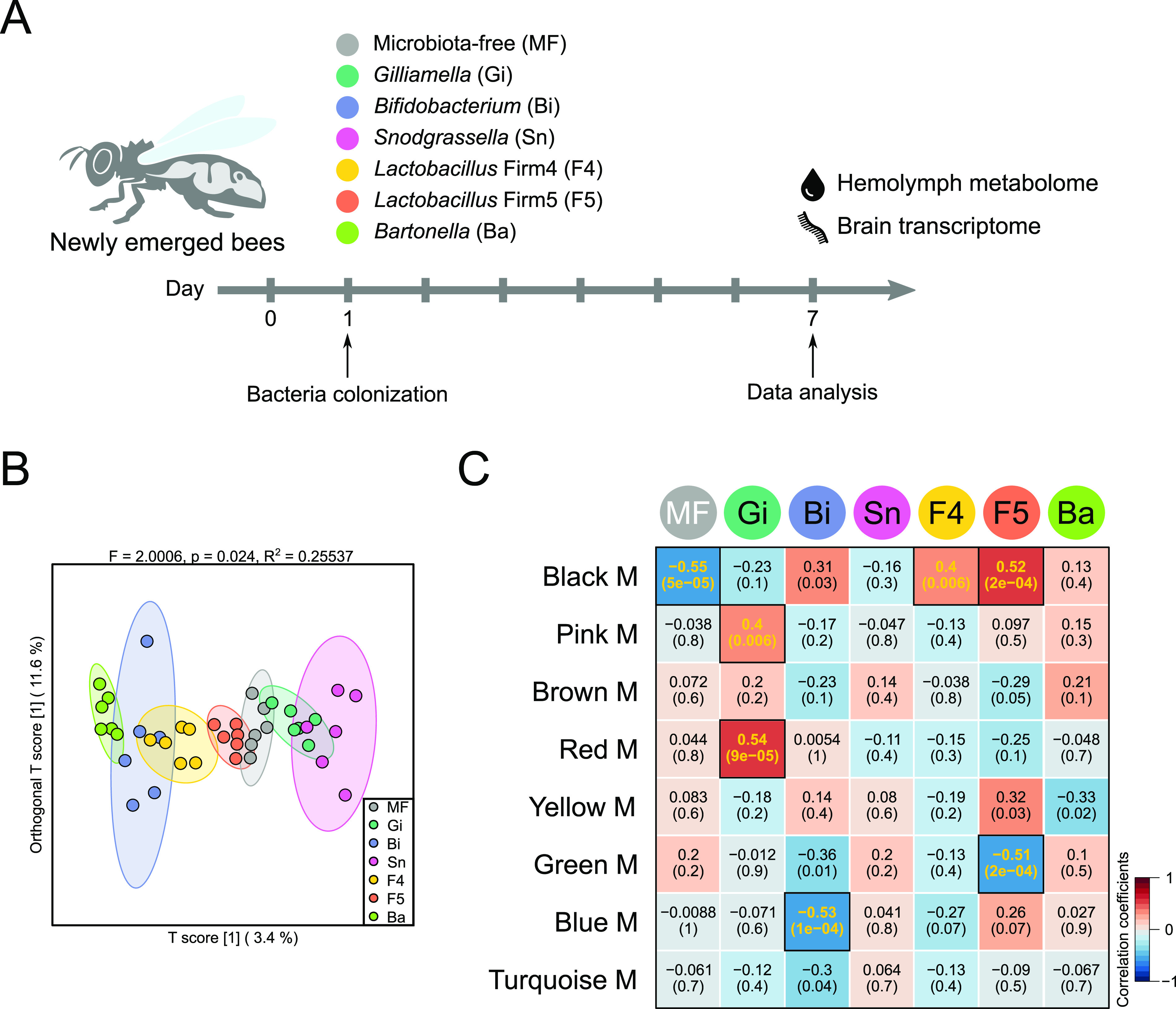
Hemolymph metabolome influenced by different honeybee gut community members. (A) Experimental design: newly emerged bees were either kept microbiota-free (MF) or mono-colonized with one bacterial isolate of each of the six species separately. Hemolymph and brain samples were collected for further analysis on day 7. (B) Orthogonal partial least squares discriminant analysis (OPLS-DA) based on all metabolites detected in the hemolymph of bees. Group differences were tested by permutational multivariate analysis of variance (PERMANOVA). (C) Weighted correlation network analysis identified eight modules (M) of metabolites highly correlated with different bee groups. Color names represent metabolite modules assigned by the WGCNA pipeline. Heatmap colors indicate positive/negative Spearman’s correlation coefficients. Correlation coefficients and *P* values are shown within the squares (yellow font, *P* < 0.01). Gi, Gilliamella apicola; Bi, Bifidobacterium asteroides; Sn, Snodgrassella alvi; F4, *Lactobacillus* Firm4; F5, *Lactobacillus* Firm5; Ba, Bartonella apis.

To reveal microbiota-induced circulating metabolome changes, we colonized newly emerged bees with six honeybee core gut bacteria, Gilliamella apicola (Gi), Bifidobacterium asteroides (Bi), Snodgrassella alvi (Sn), *Lactobacillus* Firm4 (F4) and Firm5 (F5), and Bartonella apis (Ba) (Fig. 1A and Fig. S1 in the supplemental material). Then, we performed quasi-targeted metabolomics analysis of hemolymph samples from gnotobiotic bees. In total, we identified 326 metabolites among different bee groups (Table S1 in the supplemental material). Orthogonal partial least squares discriminant analysis (OPLS-DA) showed that the metabolic signatures of hemolymph samples were significantly different between groups (Fig. 1B). To associate clusters of metabolites highly correlated with particular gut members, we performed weighted correlation network analysis (WGCNA). Bees inoculated with different gut microbes were used as the sample traits. Based on interaction patterns among the 326 metabolites, WGCNA clustered them into eight modules (M) (Table S2). Interestingly, only three modules (red, pink, and black) were significantly correlated with bees mono-colonized by *Gilliamella*, *Lactobacillus* Firm4, and *Lactobacillus* Firm5 (*P* < 0.01; Fig. 1C).

**FIG 2:**
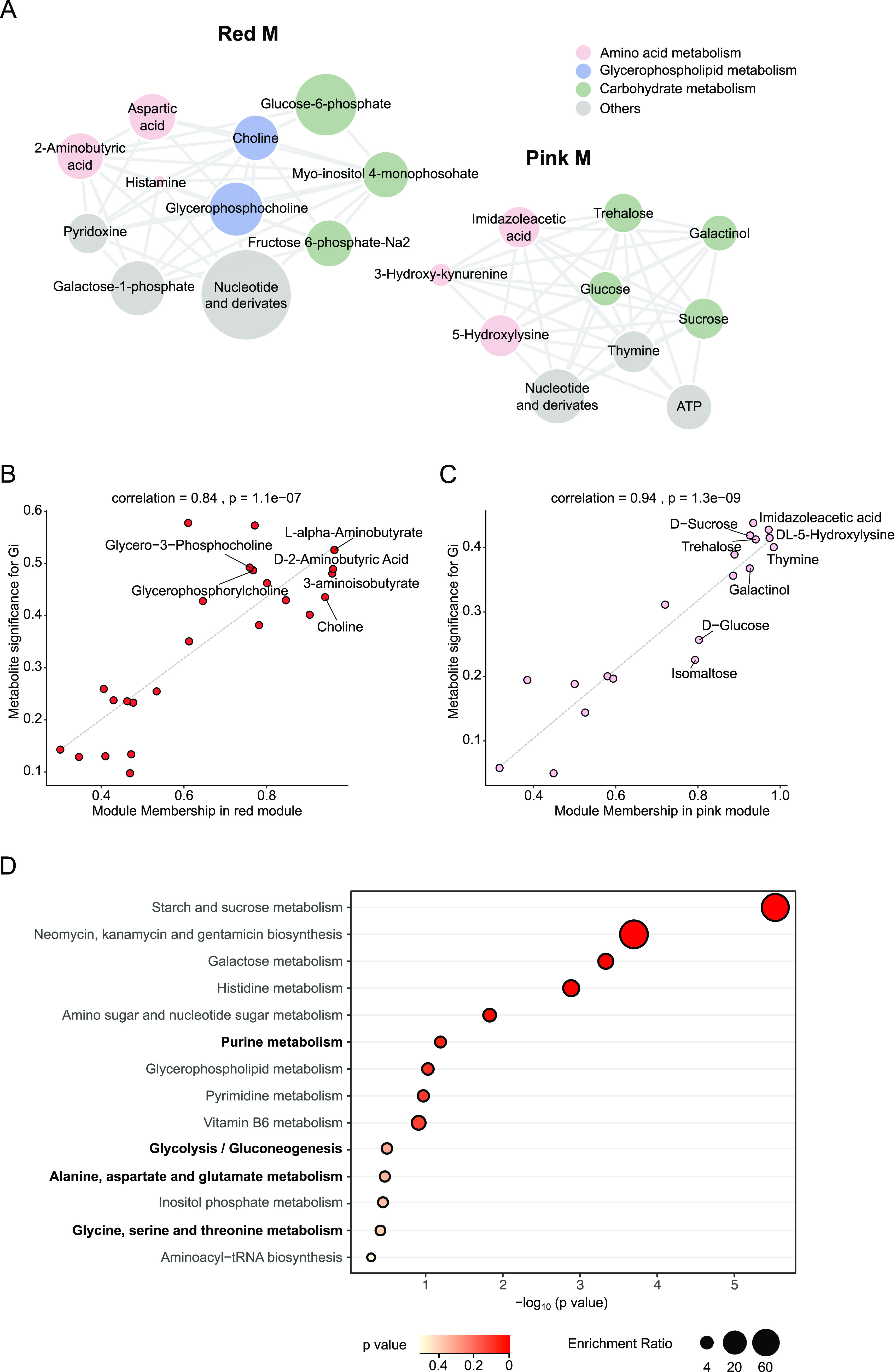
Hemolymph metabolic profile altered by *Gilliamella*. (A) Network diagrams of differential metabolites in the red and pink M. Circle colors indicate different classes of metabolites in each module. Circle size is proportional to the total abundance of metabolites in each module. (B to C) Correlation analysis between metabolite-module connectivity (*x* axis) and metabolites significantly correlated with the Gi group (*y* axis): (B) red M and (C) pink M. (D) The most significantly enriched KEGG pathways upregulated in the hemolymphs of *Gilliamella*-inoculated bees.

The relatedness of bacteria-colonized groups with significantly altered metabolites often comprises an intramodular connectivity within the network module. The red and pink M were significantly associated with the *Gilliamella* group. The metabolites that comprised the red and pink M were mainly involved in carbohydrate, glycerophospholipid, and amino acid metabolism (Fig. 2A). Specifically, the primary driving metabolites from the red M were glycerophosphocholine, choline, and glycerol-3-phosphocholine, which are involved in the glycerophospholipid metabolism pathways (Fig. 2B). In the pink M, the significantly related metabolites were sucrose, trehalose, galactinol, glucose, and isomaltose, which are enriched in carbohydrate metabolism (Fig. 2C). This is consistent with the potential of *G. apicola* for carbohydrate metabolism in the gut (17, 18). When we reanalyzed published metabolomic data obtained from the guts of mono-colonized bees (16), we found that certain pathways, including purine metabolism, glycolysis/gluconeogenesis, and alanine, aspartate, glutamate, glycine, serine, and threonine metabolism, were stimulated in both hemolymph and gut samples by the colonization of *Gilliamella* (Fig. 2D and Fig. S2A).

**FIG 3:**
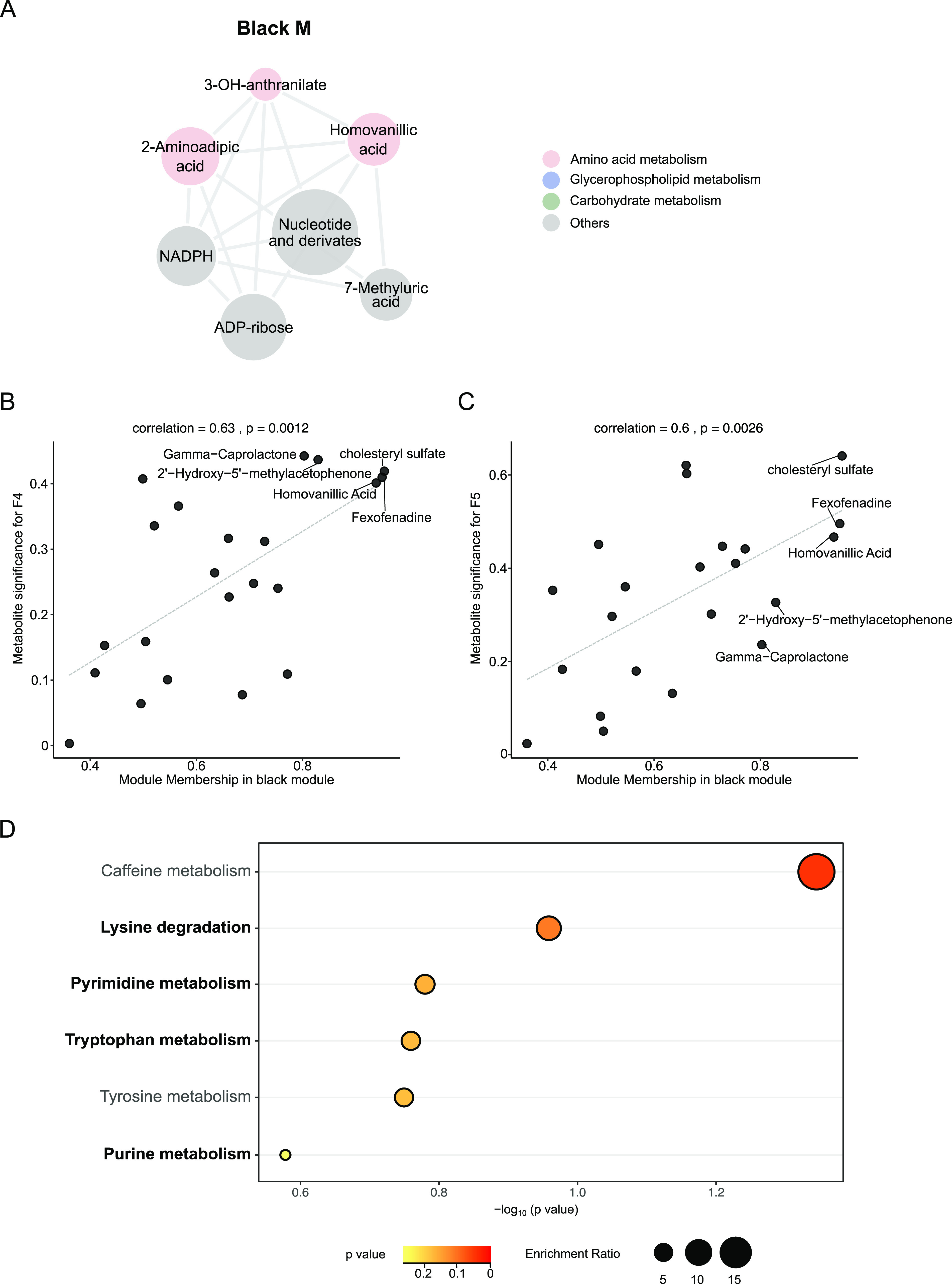
Hemolymph metabolic profile altered by *Lactobacillus* Firm4 and Firm5. (A) Network diagram of differential metabolites in the black M. Circle colors indicate different classes of metabolites in each module. Circle size is proportional to the total abundance of metabolites in each module. (B to C) Correlation analysis between metabolite-module connectivity (*x* axis) and metabolites significantly correlated with different bee groups (*y* axis): (B) black M with F4 group and (C) black M with F5 group. (D) The most significantly enriched KEGG pathways upregulated in the hemolymph of *Lactobacillus* Firm4- and Firm5-inoculated bees.

Both *Lactobacillus* Firm4 and Firm5 were significantly correlated with the black M of metabolites enriched in amino acid metabolism (Fig. 3A). Interestingly, the most-related circulating metabolite, homovanillic acid (Fig. 3B and C), is an index of brain dopamine metabolism for assessing neurologic and psychiatric illnesses, such as Parkinson’s and Huntington’s diseases (21–23). Pathways including lysine degradation and pyrimidine, tryptophan, and purine metabolism were consistently upregulated in the hemolymph and gut of F4- and F5-colonized bees (Fig. 3D and Fig. S2B–C). Altogether, these data indicate that distinctive gut members regulate specific metabolic pathways.

### Gut bacteria impact gene expression in the brain.

**FIG 4:**
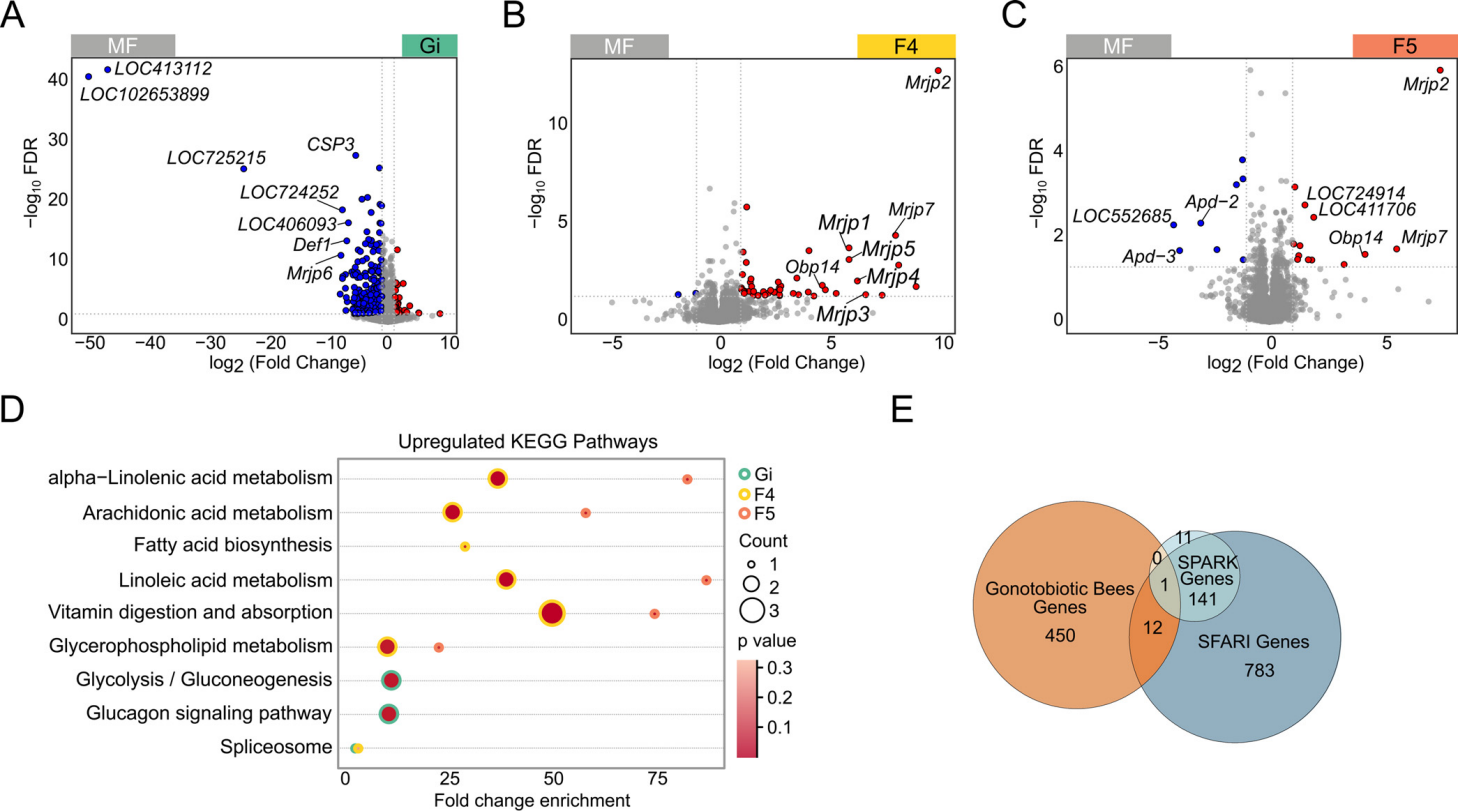
Gut microbiota impacts gene expression in the honeybee brain. (A to C) Volcano plot showing differentially regulated genes. Genes significantly enriched in bacteria-colonized bees are shown in red, and those enriched in MF bees are in blue. (D) KEGG pathways upregulated in the brains of mono-colonized bees based on differentially expressed genes. Fold change enrichment describes the proportion of genes belonging to a KEGG pathway (KO3 level) among differentially expressed genes in brains between MF and mono-colonized bees (FDR < 0.05, |log_2_-fold change| > 1). (E) Venn diagram of differentially expressed genes in brains between MF and mono-colonized bees (gnotobiotic bee genes; FDR < 0.05, Wald test with Benjamini-Hochberg correction), and their overlap with SPARK and SFARI gene data sets.

We have shown that metabolites in the circulatory system are regulated by honeybee gut bacteria. These small molecules can influence gene expression and neuronal function in the brain (24). Since the hemolymph metabolome was only modulated in bees colonized with Gi, F4, and F5, these three groups were used for brain transcriptome analysis. Our data revealed that 713 genes in total were differentially expressed in bees colonized with gut members compared to MF bees (|log_2_-fold change| >1, false discovery rate [FDR] < 0.05, Table S3), and different bee groups exhibited distinctive brain gene expression profiles (Fig. S3A). Specifically, the heat shock protein gene (*loc410620*) was upregulated in the Gi group (Fig. 4A). The odorant-binding protein gene *Obp14* was upregulated both in the F4 and F5 group (Fig. 4B and C). Insect odorant-binding proteins are essential for detection and distinguishing of specific odors (25). In addition, several *mrjp* family genes of the major royal jelly protein (MRJP) encoded in the *A. mellifera* genome were significantly upregulated in the F4 and F5 groups, while bees colonized with *Gilliamella* exhibited decreased expression of *mrjp* genes (Fig. 4A–C). MRJPs have polyfunctional properties and participate in all major aspects of eusocial behavior in honeybees, such as caste determination and age polyethism (26).

Enrichment analysis of differentially expressed genes determined that KEGG pathways, including those for alpha-linolenic, arachidonic acid, linoleic, vitamin, and glycerophospholipid metabolism, were upregulated in the brains of F4 and F5 groups (Fig. 4D). The glycolysis/gluconeogenesis and glucagon signaling pathway, which is critical for brain physiology as it provides the fuel for brain functions (27), was only upregulated in bees colonized with *Gilliamella*. A KEGG pathway involving RNA processing by the spliceosome was upregulated in Gi and F4 bees. These results showed that the transcriptomic programs were differentially altered in the bacteria-colonized groups.

We then performed Pearson correlation analysis to analyze the relationship between gene expression and the metabolites which are significantly regulated by Gi, F4, and F5 separately (Fig. S3B–D). In the Gi group, carbohydrate metabolites showed significantly positive correlations (*P* < 0.05) with upregulated genes in the brain, while metabolites involved in glycerophospholipid metabolic pathways showed significantly negative correlations with those genes (Fig. S3B). In the F4 and F5 groups, fexofenadine, cholesteryl sulfate, and 2′-hydroxy-5′-methylacetophenone showed significantly positive correlations with upregulated genes in the brain, while homovanillic acid and gamma-caprolactone showed significantly negative correlations with those genes (Fig. S3C–D). These results suggest that upregulated genes in the brain were correlated with specific metabolites altered by different gut bacteria, indicating the potential roles of circulating metabolisms in host-bacteria interactions.

### Gut bacteria impacts alternative splicing of genes in the brain.

Shpigler et al. (28) have reported that the gene expression signatures of honeybees with disordered social behaviors are significantly enriched for human autism spectrum disorder (ASD)-related genes. Likewise, the differentially expressed genes in bacteria-colonized bees overlapped with those from human ASD patients (Fig. 4E), implying the involvement of gut microbiota in host behaviors. However, the differentially expressed genes present in the SFARI data set did not overlap the gene set for bees with disordered behaviors, as defined by Shpigler et al. (28).

Voineagu et al. (29) have shown that dysregulation of alternative splicing (AS) in ASD-related genes is also associated with the psychiatric disorder. Given evidence for spliceosome alterations, we investigated whether gut bacteria-colonized bee brains showed different AS events compared with those of MF bees. rMATS analysis of alternative splicing events of brain genes detected a total of 18,985 events in 4,929 genes, and skipped exon (SE) was the most abundant of the different types of AS. About 10 to 25% of events for each type of AS showed significantly different inclusion rates in bacteria-colonized bees (Fig. S4A). The relative abundance of different AS event types was similar across bee groups (Fig. S4A). However, the UpSet plot shows that the vast majority of events do not intersect between sets, indicating that multiple AS events can occur in a single gene and that the gut members cause different events (Fig. S4B).

**FIG 5:**
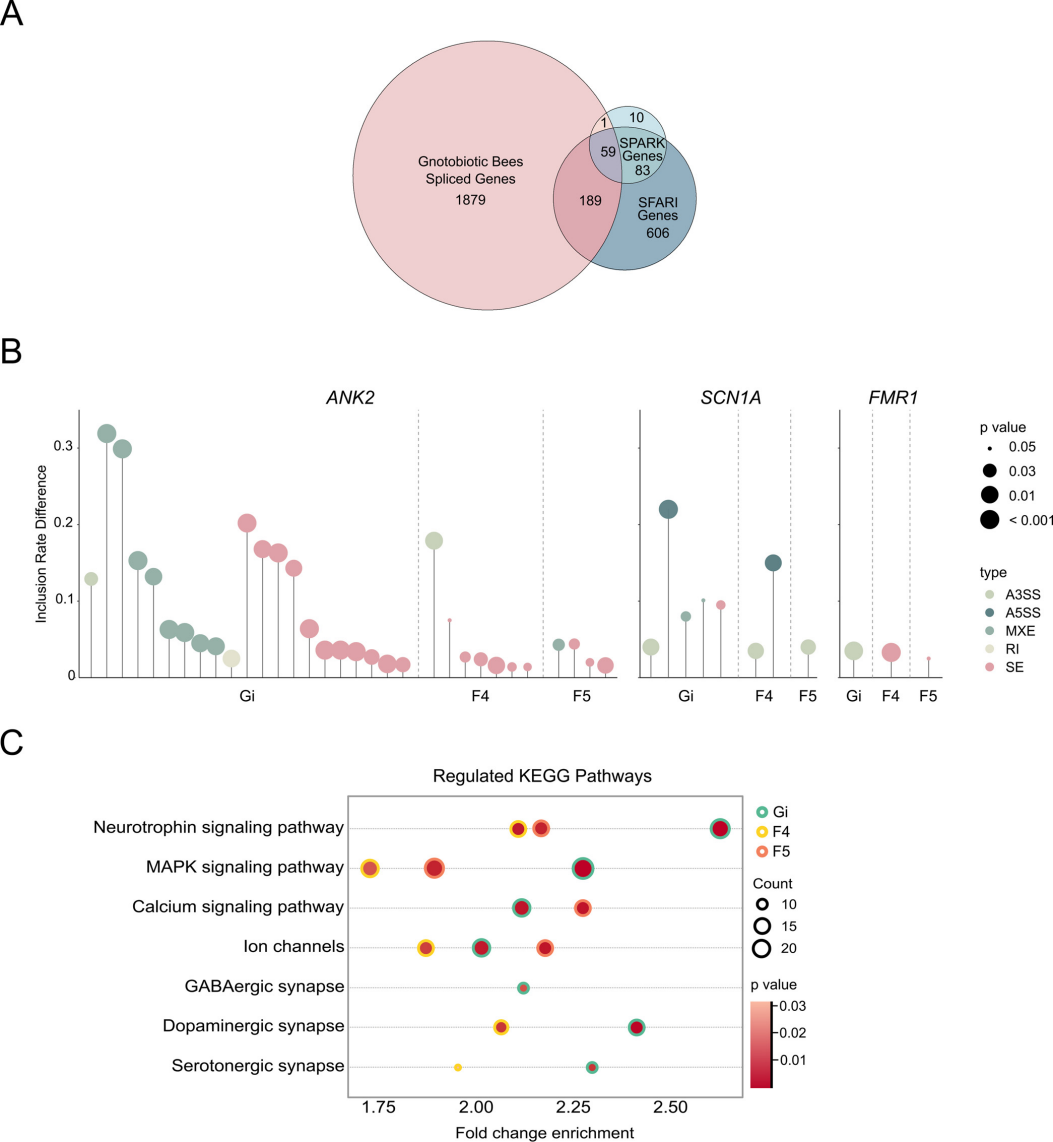
Gut microbiota impacts alternative splicing of high-confidence ASD genes in the honeybee brain. (A) Venn diagram of differentially spliced genes in the brains between MF and mono-colonized bees (gnotobiotic bee spliced genes; *P* < 0.05), and their overlap with SPARK and SFARI gene data sets. (B) Differentially splicing events (*P* < 0.05) in *Ank2* present in both SPARK and SFARI gene data sets. Differential splicing events were identified by rMATS. A3SS, alternative 3′ splice site; A5SS, alternative 5′ splice site; MXE, mutually exclusive exon; RI, retained introns; SE, skipped exon. (C) KEGG pathways regulated in the brains of mono-colonized bees based on differentially spliced genes. Fold change enrichment describes the proportion of genes belonging to a KEGG pathway (KO3 level) among differentially spliced genes in the brains between MF and mono-colonized bees (gnotobiotic bee spliced genes; FDR < 0.05, |log_2_-fold change| > 1).

Next, we examined the overlap of genes showing significantly differential AS events between MF and bacteria-colonized bees with the ASD risk genes from the SPARK and SFARI gene data sets (30). Fifty-nine of the 2,128 differentially spliced genes in MF bees are associated with human autism (Fig. 5A). Interestingly, almost all identified homologs belong to the high-confidence SFARI gene list (category 1) implicated in ASD (Table S4). Specifically, genes related to the pathophysiology of ASD are differentially spliced in MF bees compared with bacteria-colonized bees. For example, genes belonging to the functional terms of postsynaptic membrane (*Ank2*, *Fmr1*) and voltage-gated ion channel (*Scn1a*) showed different inclusion rates, and these are mostly regulated in the Gi groups compared to in the MF bees (Fig. 5B). Taking these findings together, we identified that gut microbes induce differential gene expression profiles and mediate AS, resulting in specific gene isoforms in the honeybee brain. The genes essential for bee social behaviors and related to human ASD disease are affected especially by *Gilliamella*, confirming the similarities of genes associated with social responsiveness in humans and honeybees (28).

### Gut microbiota alters brain neurotransmitter level.

**FIG 6:**
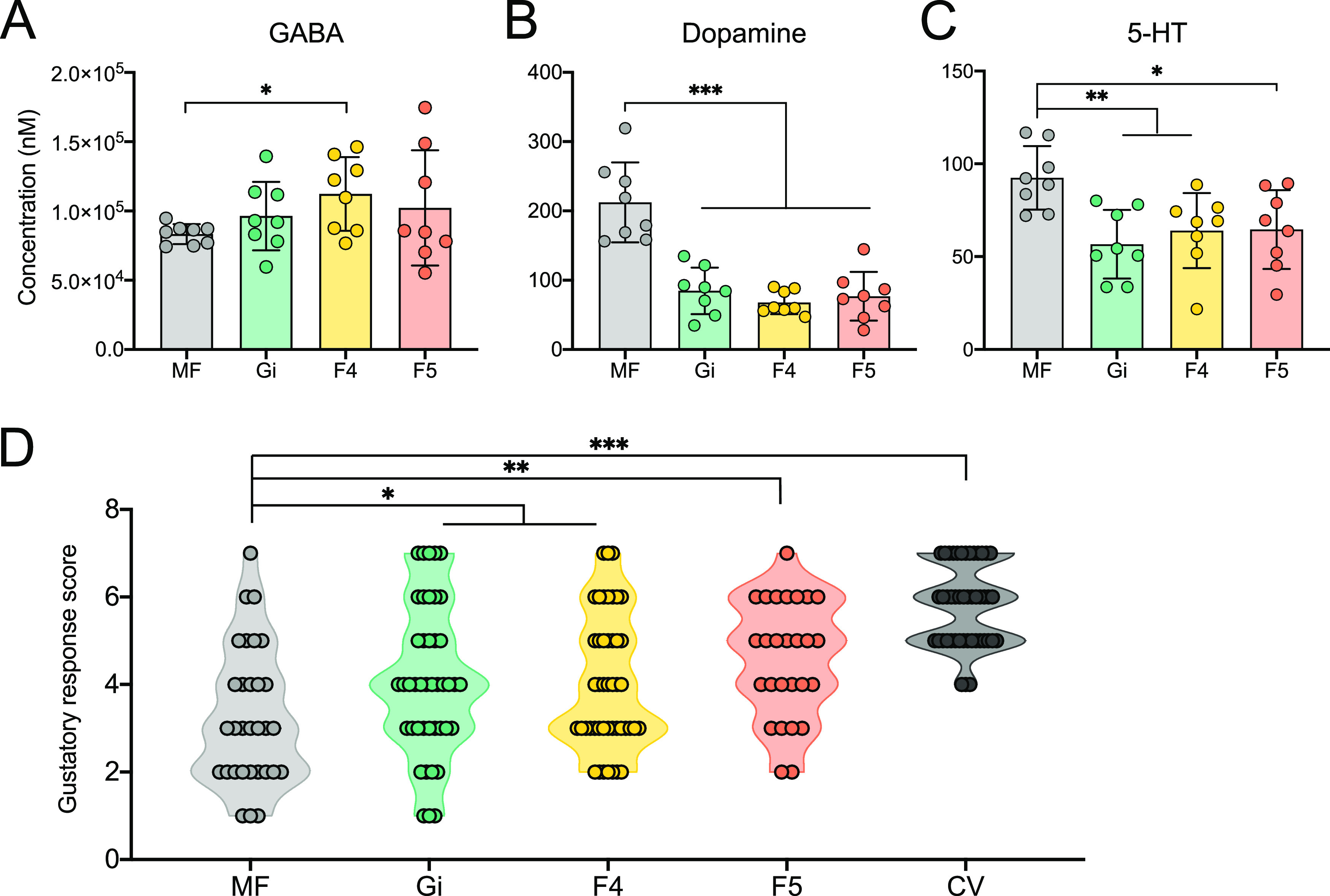
Gut microbiota alters the concentration of neurotransmitters in the brain and honeybee behavior. (Ato C) Concentrations of (A) GABA, (B) dopamine, and (C) 5-HT in the brains of MF (*n* = 8), Gi (*n* = 8), F4 (*n* = 8), and F5 (*n* = 8) bees. (D) Distribution of gustatory response scores of MF bees (*n* = 31) and bees mono-colonized with different core gut bacteria: Gi (*n* = 43), F4 (*n* = 46), F5 (*n* = 27), and conventional bees (CV, *n* = 46). Each circle indicates a bee response to the provided sucrose concentration. Differences between bacteria-colonized and MF bees were tested by Mann-Whitney U test (*, *P* < 0.05; **, *P* < 0.01; ***, *P* < 0.001).

The enrichment analysis of differentially spliced genes identified that KEGG pathways, including the neurotrophin, MAPK, and calcium signaling pathways, as well as ion channels and the GABAergic, dopaminergic, and serotonergic synapses, were regulated in the brains of the Gi, F4, and F5 groups (Fig. 5C). Therefore, we investigated changes in the brain neurochemistry of MF and mono-colonized bees. The concentrations of three major neurotransmitters, GABA, 5-HT, and dopamine, which are important modulators of honeybee feeding behavior, were determined (31–33). The concentration of the inhibitory transmitter GABA was significantly higher in the brains of F4 bees (Fig. 6A). In contrast, the concentrations of dopamine and 5-HT were significantly lower in bees mono-colonized with Gi, F4, and F5 than in the MF bees (Fig. 6B and C).

### Gut bacteria affects sucrose responsiveness.

So far, our results have illustrated the impact of gut bacteria on the brain transcriptomic profile and neurochemistry of the honeybee. Finally, we tested whether the colonization of specific gut species alters host behavior. Proboscis extension response (PER) is a taste-related behavior that is fundamental for olfactory discrimination and colony performance in honeybees (34). We then measured the PER of MF bees, conventional (CV) bees, and bees mono-colonized with Gi, F4, and F5 to determine whether specific gut members influence host behavior. Compared with that of the MF group, sucrose sensitivity was significantly elevated in bacteria-colonized bees (Fig. 6D). Interestingly, CV bees were more sensitive to low sucrose concentrations compared to mono-colonized bees, implying an integrative effect of gut bacteria on honeybee behavior. Our findings indicate that colonization of either the normal gut microbiota or single core gut member can affect neurotransmitter levels in the brain, which might be associated with altered olfactory sensitivity.

## DISCUSSION

The influence of gut community members on the host is mainly driven by the microbial metabolism, specifically the amino acid, lipid, and carbohydrate metabolic pathways, which can further influence the circulation and synthesis of neuroactive molecules in the host. We found that distinct gut members regulated different sets of metabolites in the hemolymph and intestine. These specialized bee gut bacteria with distinct functions might be the results of long-term coevolution with the host (35), which provides an optimized benefit to the host. Specifically, *Gilliamella* regulate host carbohydrate metabolism, which consistent with its capability to digest mono- and polysaccharides in the bee gut (18, 36). *Lactobacillus* Firm4 and Firm5 were significantly correlated with amino acids. Among these, homovanillic acid, an indicator of brain dopamine metabolism for assessing neurologic and psychiatric illness (21–23), was mostly regulated, corroborating with the altered dopamine level in the brain. Similar gut-brain communication has been recently demonstrated in *Drosophila*. For example, the gut symbiont of *Drosophila* modulates trehalose levels through xylose isomerase, resulting in downregulated octopamine production and decreased locomotor behavior (37). Oral infection with Erwinia carotovora can stimulate the production of gut-derived Upd proteins, which further promote lipid production and accumulation in neurons and modulate olfaction in aging flies (38).

The homologous molecular mechanism of social responsiveness between honeybees and humans has been well documented (28). Bees with disordered social behaviors show a transcription profile in the brain which is distinct from that of normal bees. The differentially expressed genes in unresponsive individuals are enriched for human ASD-related genes. In the halictid bee *Lasioglossum albipes*, these genes are also regulated in the solitary individuals compared to those in social populations, indicating their implications in social behaviors (39). Despite the disturbed gene expression level, the aberrant alternative splicing of ASD-related genes is also involved in mental disorders (40). In our data set, the analysis of alternative splicing of genes identified differences between bacteria-colonized bees. The altered genes compared to those of MF bees overlapped with the SFARI gene data set for autism and those associated with disordered bee social responsiveness (Fig. 5). The mutually exclusive exon (MXE) and SE events of a high-confidence ASD risk gene, *Ank2*, were predominantly affected by the gut bacteria, especially *Gilliamella*. This gene is also affected in the brains of mice colonized by ASD-human gut microbiota (40). Correspondingly, our enrichment analysis of differentially expressed genes revealed that the KEGG pathway related to spliceosome was upregulated in the Gi and F4 groups, supporting the contribution of gut bacteria to splicing regulation in the brain. All these findings demonstrate the deep conservation of genes related to social responsiveness in humans and distantly related insect species, indicating the potential role of gut microbes in social evolution (41).

Neurotransmitters that carry and pass information between neurons are essential for brain functions, which are important modulators of behaviors. In honeybees, four monoamine neurotransmitters play important roles in olfactory sensitivity (31–33). Specifically, dopamine and 5-HT inhibit appetitive learning and decrease sucrose sensitivity in foragers (31, 32). Also, GABA and acetylcholine have been physiologically characterized as inducing currents between neurons within the olfactory pathways and contributing to odor memory formation (42). The inhibitory transmitter GABA is also required for fine odor discrimination (33). The concentrations of most identified neurotransmitters were regulated by different gut members, corroborating with the roles of gut microbiota in altered behavior. Notably, we only analyzed the concentrations of GABA, dopamine, and 5-HT in this study, although acetylcholine is also pivotal for the integration of sensory information in honeybees. Specifically, cholinergic signaling via muscarinic receptors is critical for olfactory associative learning and foraging behaviors (43). Moreover, stimulation of the muscarinic receptor in the honeybee increases the volume of the mushroom body neuropil, which mimics the reinforcement of cholinergic neurotransmission in foraging bees (44). A reduced mushroom body calycal growth has also been associated with lower learning performance in bumblebees through microcomputed tomography scanning (45). It would be interesting to investigate whether gut microbes impact structural changes of the brain in future studies.

Although mono-colonization is sufficient to affect host behaviors, ecological interactions in bacterial communities cannot be neglected. In the honeybee gut, individual community members occupy different metabolic niches which contribute to the overall output of an integrative gut microbiota (16). In particular, cross-feeding exists between *Gilliamella* and *Snodgrassella*, which may form a symbiotic network for the utilization of nutrient resources (20). Different strains of *Bifidobacterium* and *Gilliamella* cooperate to digest diet polysaccharides for the honeybee host (18). Interactions between the main species in the gut enable efficient substrate metabolism as well as community stability, thus benefiting the host. We observed that the CV bees showed the highest gustatory responsiveness, which indicates that potential interactions of gut members contribute to the host physiology (Fig. 6D). Thus, we hypothesized that the coexistence and interaction of bacteria in host-associated microbial communities have a greater impact on host physiology and behavior than that of individual community members. In addition, we studied honeybees mono-colonized with single bacterial strains, which is a gut dysbiosis to some extent. In consistent with the findings for taste-related behavior, mono-colonized bees exhibited inferior performance in a PER assay compared with CV bees. Further experiments using an artificial community with defined gut members may advance our understanding of the actual roles of different bacteria in the microbiome.

In summary, our mono-colonization experiments provide unprecedented insights into the impact of honeybee gut bacteria on host behavior. In particular, we disentangled the contributions of different community members to the host, from circulating the metabolome to transcriptional and neurochemical changes in the brain. Our results highlight the important roles of gut microbiota in honeybee behaviors and the complex interactions of different bee gut members contributing to the host physiology.

## MATERIALS AND METHODS

### Generation of microbiota-free and mono-colonized honeybees.

Microbiota-free bees were obtained as described by Zheng et al. (18) with modifications (Fig. S1). Late-stage pupae were removed manually from brood frames and placed in sterile plastic bins. The pupae emerged in an incubator at 35°C, with humidity of 50%. Newly emerged MF bees (day 0) were kept in axenic cup cages with sterilized sucrose syrup (50%, wt/vol) for 24 h and divided into three groups: (i) MF, (ii) mono-colonized (MC), and (iii) CV bees. For each setup, 20 to 25 MF bees (day 1) were placed into one cup cage and fed on the corresponding solutions or suspensions for 24 h. For the MF group, 1 mL of 1× phosphate-buffered saline (PBS) was mixed with 1 mL of sterilized sucrose solution (50%, wt/vol) and 0.3 g sterilized pollen. For the MC group, stocks of Gilliamella apicola (W8127), Snodgrassella alvi (W6238G3), Bifidobacterium asteroides (W8113), Bartonella apis (B10834G6), *Lactobacillus* Firm4 (W8089), and *Lactobacillus* Firm5 (W8172) in 25% glycerol stock at −80°C were resuspended in 1 mL 1×PBS (Solarbio, Beijing, China) at a final optical density at 600 nm (OD_600_) of 1, and then mixed with 1 mL sterilized sucrose solution (50%, wt/vol) and 0.3 g sterilized pollen. For the CV group, 5-μL homogenates of freshly dissected hindguts of nurse bees from their hives of origin were mixed with 1 mL 1×PBS, 1 mL sterilized sucrose solution (50%, wt/vol), and 0.3 g sterilized pollen. Then, MF, MC, and CV bees were provided sterilized sucrose (0.5 M) with sterile pollen and kept in an incubator (35°C, RH 50%) until day 7. Brains, guts, and hemolymph of bees were collected on day 7 for further analysis. All bees used for behavior experiments and tissue collection came from the same colony. Bees coming from the same cup cage for each group were considered a single replicate. Here, we used *n* = 3 replicates per group, 20 to 30 bees per replicate. Colonization levels of MF and MC bees were determined by CFU from dissected guts, as described by Kwong et al. (46).

### Tissue collection.

Whole guts were dissected by tweezers sterilized with 75% alcohol. Dissected guts were directly crushed in 25% (vol/vol) glycerol on ice for bacterial load quantification or collected into an empty 1.5-mL centrifuge tube for metagenomic sequencing and metabolomics analysis. All gut samples were frozen at −80°C until analysis. Honeybee brains were collected using a dissecting microscope (Canon). Individual bees were fixed on beeswax using two insect needles through the thorax. After removal of the head cuticle, the whole brain was placed on a glass slide and soaked in RNAlater (Thermo; Waltham, MA, USA) or proteinase inhibitor (Roche; Mannheim, Germany) for gene expression profiling, proteome analysis, and neurotransmitter concentration quantification. Then, the hypopharyngeal glands, salivary glands, three simple eyes, and two compound eyes were carefully removed. Dissected brains were kept frozen at −80°C. Hemolymph was collected using a 10-μL pipettor (Eppendorf; Hamburg, Germany) from an incision above the median ocellus. A minimum of 50 μL hemolymph was collected from 10 bees into a 1.5-mL centrifuge tube. During the collection process, tubes were temporarily preserved on dry ice and subsequently stored at −80°C until analysis. Brain and hemolymph samples were collected from different individual bees following the same colonization step.

### Quasi-targeted metabolomics analysis.

Hemolymph metabolites were determined by quasi-targeted metabolomics using high-pressure liquid chromatography (HPLC)-tandem mass spectrometry (MS-MS). First, 50 μL of hemolymph samples were mixed with 400 μL prechilled methanol by vortexing. All samples were incubated on ice for 5 min and then centrifuged at 15,000 × *g* at 4°C for 10 min. The supernatant was diluted to a final concentration containing 53% methanol by liquid chromatography-mass spectrometry (LC-MS) grade water. The samples were then transferred to a fresh vial and centrifuged at 15,000 × *g* at 4°C for 20 min. Finally, the supernatant was injected into the LC-MS-MS system, and the analyses were performed using an ExionLC AD system (SCIEX) coupled with a QTRAP 6500+ mass spectrometer (SCIEX). Samples were injected onto a BEH C8 column (100 mm × 2.1 mm × 1.9 μm) using a 30-min linear gradient at a flow rate of 0.35 mL/min for the positive-polarity mode. Eluent A was 0.1% formic acid-water, and eluent B was 0.1% formic acid-acetonitrile. The solvent gradient was set as follows: 5% B, 1 min; 5 to 100% B, 24.0 min; 100% B, 28.0 min; 100 to 5% B, 28.1 min; 5% B, 30 min. The QTRAP 6500+ mass spectrometer was operated in positive-polarity mode with curtain gas at 35 lb/in^2^, collision gas at medium, ion-spray voltage at 5,500 V, temperature at 500°C, ion source gas at 1:55, and ion source gas at 2:55. For negative-ion mode, samples were injected onto aHSS T3 Column (100 mm × 2.1 mm) using a 25-min linear gradient at a flow rate of 0.35 mL/min. The solvent gradient was set as follows: 2% B, 1 min; 2 to 100% B, 18.0 min; 100% B, 22.0 min; 100 to 5% B, 22.1 min; 5% B, 25 min. The QTRAP 6500+ mass spectrometer was operated in negative polarity mode with curtain gas at 35 lb/in^2^, collision gas at medium, ion-spray voltage at −4,500 V, temperature at 500°C, ion source gas at 1:55, and ion source gas at 2:55.

Detection of the experimental samples using MRM was based on the Novogene in-house database. Q3 (daughter) was used for the quantification. Q1 (parent ion), Q3, retention time, declustering potential, and collision energy were used for metabolite identification. Data files generated by HPLC-MS/MS were processed with SCIEX OS (version 1.4) to integrate and correct the peaks. A total of 326 compounds were identified in the hemolymph samples. Metabolomics data analysis was then performed using MetaboAnalyst 4.0 (47).

### Weighted gene coexpression network analysis.

R software package WGCNA 1.69 (48) was used to identify key phenotype-related metabolic modules based on correlation patterns. The Pearson correlation matrix was calculated for all possible metabolite pairs and then transformed into an adjacency matrix with soft thresholding power set to 5 for the best topological overlap matrix. A dynamic tree cut algorithm was used to detect groups of highly correlated metabolites. The minimum module size was set to 14, and the threshold for merging module was set to 0.25 as default. Each module was assigned a unique color and contained a unique set of metabolites. After modules had been obtained from each group, module eigenmetabolite was calculated with the “ModuleEigengenes” function. Association analysis between a module and the trait of each group was performed using the “corPvalueStudent” function based on the module eigenmetabolite. Statistical significance was set at *P* < 0.01. Metabolites in each module were annotated on the KEGG database and classified into major categories using MetaboAnalyst 4.0 (47) for enrichment analysis. Finally, the network connections among metabolites in modules were visualized using Cytoscape 3.7.0 (49).

### Brain gene expression analysis.

Total RNA was extracted from individual brains using the Quick-RNA MiniPrep kit (Zymo; Irvine, CA, USA). RNA degradation and contamination were monitored on 1% agarose gels, and the purity was checked with the NanoPhotometer spectrophotometer (IMPLEN; CA, USA). RNA integrity was assessed using the RNA Nano 6000 assay kit of the Bioanalyzer 2100 System (Agilent Technologies; Santa Clara, CA, USA). RNA sequencing libraries were generated using the NEBNext Ultra RNA Library Prep Kit for Illumina (New England BioLabs; Ipswich, MA, USA), and index codes were added to attribute sequences to each sample. Clustering of the index-coded samples was performed on the cBot Cluster Generation System using a TruSeq PE Cluster Kit v3-cBot-HS (Illumina; San Diego, CA, USA), and the library preparations were then sequenced on an Illumina NovaSeq 6000 platform (Illumina; San Diego, CA, USA) and 150-bp paired-end reads were generated.

The sequencing quality of individual samples was assessed using FastQC version 0.11.5 with default parameters. An index of the bee reference genome (Amel_HA version 3.1) was built using HISAT2 version 2.0.5 (50), and the FastQC trimmed reads were then aligned to the built index using HISAT2 version 2.1.0 with default parameters. Gene expression was quantified using HTSeq version 0.7.2 (51) with ‘union’ mode, and only reads mapping unambiguously to a single gene were counted. In contrast, reads aligned to multiple positions or overlapping more than one gene were discarded. If it were counted for both genes, the extra read from the differentially expressed gene might have caused the other gene to be wrongly identified as differentially expressed, so we chose ‘union’ mode.

Differential gene expression analysis was performed using the DESeq2 package (52) in R. We modeled read counts following a negative binomial distribution with normalized counts and dispersion. The proportion of the gene counts in the sample to the cDNA concentration was scaled by a normalization factor using the median-of-ratios method. The variability between replicates was modeled by the dispersion parameter using empirical Bayes shrinkage estimation. For each gene, we fit a generalized linear model to obtain the overall expression strength of the gene and the log_2_-fold change between the MC and MF groups. For significance testing, differential gene expression was determined by the Wald test. The resulting *P* values were corrected for multiple comparisons using the Benjamini-Hochberg FDR method (53). Genes with an adjusted *P* value of <0.05 and a |log_2_-fold change| of >1 were assigned as differentially expressed.

To get a better annotation of the honeybee reference genome, we reannotated it using eggNOG-mapper version 5.0 (54). A total of 6,269 out of 12,375 honeybee genes were successfully assigned to a KO entry with ‘diamond’ mode, and hierarchy information for the KEGG metabolic pathway was extracted. Functional analysis of differentially expressed genes was performed based on KEGG Orthologue (KO) markers. The percentages of KO markers belonging to each category (KEGG class at level 3) out of the total MC- and MF-enriched KO markers were designated as comparison parameters. The significance level was calculated by Fisher’s exact test using clusterProfiler version 3.10.1 (55). Correlation analysis between regulated hemolymph metabolites and upregulated genes was performed using Pearson’s correlation analysis. The correlation coefficients and the corresponding *P* values were calculated in R.

Analysis of event-level differential splicing was performed using rMATS version 4.0.2 (56) based on the bee reference genome. An exon-based ratio metric, commonly defined as percent-spliced-in (PSI) value, was employed to measure the alternative splicing events. An effective length of l was used for normalization. The PSI value was calculated for several classes of alternative splicing events, including skipped exon (SE), alternative 5′ splice site (A5SS), alternative 3′ splice site (A3SS), mutually exclusive exons (MXE), and retained introns (RI). Events with a *P* of <0.05 were considered to be differentially spliced across gnotobiotic bees and microbiota-free bees.

To find overlaps between the differentially expressed or spliced genes of bee brain and those from human autism spectrum disorders, a total of 3,531 high-quality reference protein sequences corresponding to 948 known autism risk genes (SFARI: https://gene.sfari.org/, SPARK for autism: http://spark-sf.s3.amazonaws.com/SPARK_gene_list.pdf) were aligned against protein sequences of the honeybee genome using BLASTP (57) with a two-way best matching strategy. In total, 649 autism protein sequences obtained a match (similarity > 30% and E value < 0.000394). Then we calculated the intersection of the autism risk genes and the differentially expressed or spliced genes between bacteria-colonized and MF bees (*P* < 0.05).

### Targeted metabolomics for brain neurotransmitters.

Brain tissues dissected from MF and MC bees were sent to Biotree Biotech Co. Ltd. (Shanghai, China) for targeted metabolomics analysis of GABA, dopamine, and serotonin. Six brain tissues from one treatment group were put into one tube and centrifuged (2,400 g × 1 min at 4°C). A 100-μL volume of acetonitrile containing 0.1% formic acid and 20 μL ultrapure water was added and the tubes were vortexed thoroughly. Metabolites were sonicated in an ice-water bath for 30 min, followed by subsiding at −20°C for 2 h. Supernatants were collected after centrifugation (14,000 g × 10 min at 4°C). Next, 20 μL of supernatant was transferred to a new vial followed by incubation for 30 min after the addition of 10 μL sodium carbonate solution (100 mM) and 10 μL 2% benzoyl chloride acetonitrile. Then, 1.6 μL internal standard and 20 μL 0.1% formic acid was added and the samples were centrifuged (14,000 g × 5 min at 4°C). A total of 40 μL of the supernatants was transferred to an auto-sampler vial for downstream UHPLC-MS/MS analysis. Serotonin hydrochloride, γ-aminobutyric acid, and dopamine hydrochloride (Aladdin Bio-Chem Technology; Shanghai, China) derivatized with benzoyl chloride (Sigma-Aldrich; Saint Louis, MO, USA) were used for the construction of the calibration standard curve. The internal standards mixtures (γ-aminobutyric acid, dopamine hydrochloride, and serotonin hydrochloride) derivatized with benzoyl chloride-d5 (Sigma-Aldrich; Saint Louis, MO, USA) (58) of the corresponding concentrations were prepared.

The UHPLC separation was carried out using an ExionLC System (AB SCIEX; Framingham, MA, USA), and samples were analyzed on the QTRAP 6500 LC-MS/MS system (AB SCIEX). Two μL of samples was directly injected onto an ACQUITY UPLC HSS T3 column (100 mm × 2.1 mm × 1.8 μm; Waters Corporation; Milford, Ma, USA). The column temperature was set at 40°C, and the auto-sampler temperature was set at 4°C. Chromatographic separation was achieved using a 0.30 mL/min flow rate and a linear gradient of 0% to 2% B in 2 min; 2 to 98% B in 9 min, 98% B for 2 min, and equilibration for 2 min. Solvent A was 0.1% formic acid and solvent B was acetonitrile. For all multiple-reaction monitoring (MRM) experiments, 6500 QTrap acquisition parameters were as follows: ion-spray voltage at 5,000 V, curtain gas setting of 35, nebulizer gas setting of 60, and temperature at 400°C. Raw data were analyzed using Skyline (59).

### Gustatory responsiveness.

Seven-day-old MF, MC, and CV bees were used to measure responses to different concentrations of sucrose solution as previously described, with some modifications (9). Before the test, bees were starved for 2 h in the incubator by removing sugar syrup and bee bread from the cup cage. Bees were then mounted to modified 2.0-mL centrifuge tubes using Parafilm M (Bemis; Sheboygan Falls, WI, USA), and they could only move their heads and propodeum for antennae sanitation. Individual responsiveness was measured by presenting a series of sucrose solution concentrations (0.1, 0.3, 1, 3, 10, 30, and 50%; wt/vol) to the antennae of bees (60). Before each sucrose solution presentation, all bees were tested for their response to pure water to control for the potential effects of repeated sucrose stimulations, which could have led to either sensitization or habituation (61). The interstimulus interval between water and sucrose solution was 4 min. When a bee’s antenna is stimulated with a sucrose solution of sufficient concentration, the bee reflexively extends its proboscis. The lowest sucrose concentration at which an individual responded by extending its proboscis was recorded and interpreted as its sugar response threshold. At the end of the experiment, a gustatory response score was obtained for each bee based on the sucrose concentrations to which the bees responded. The response was arbitrarily quantified with scores of 1 to 7, where 1 represented a bee that only responded to the highest sucrose concentration and 7 represented an individual that responded to all concentrations tested. Bees that did not respond to any of the sucrose concentrations were excluded from further analyses. Additionally, bees that responded to all concentrations of sucrose solutions and all water presentations were also excluded, as they appeared to be unable to discriminate between sucrose and water (61).

### Data availability.

Raw sequence reads have been deposited in the NCBI SRA database under the BioProject accession no. PRJNA783076.
