# Supplementary material for: Distinct Roles of Honeybee Gut Bacteria on Host Metabolism and Neurological Processes
Source: Microbiol Spectr. 2022 Mar 10;10(2):e02438-21. doi: 10.1128/spectrum.02438-21 (PMC9045319; doi:10.1128/spectrum.02438-21)
Supplement: SUPPLEMENTAL FILE 1 — Supplemental material. Download SPECTRUM02438-21_Supp_1_seq10.pdf, PDF file, 3.4 MB [file spectrum02438-21_supp_1_seq10.pdf]

Supplemental Material

For: **Distinct roles of honeybee gut bacteria on host metabolism and neurological process**

Zijing Zhang, Xiaohuan Mu, Yao Shi, Hao Zheng,

Contents **Page**

Contents	Page
Figure S1	2
Figure S2	3
Figure S3	4
Figure S4	5
Table S1	6
Table S2	6
Table S3	6
Table S4	6

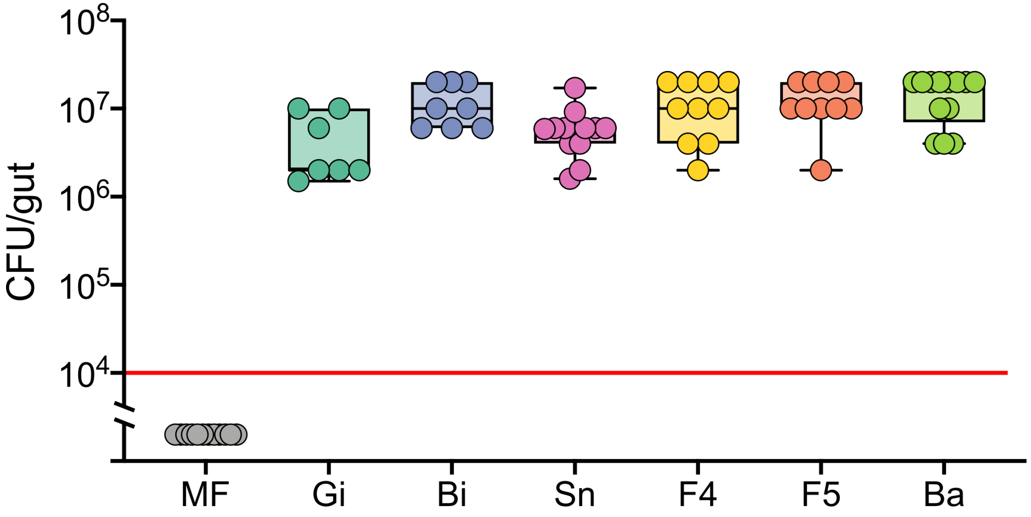

FIG S1 Boxplots of the total CFU per gut estimated by bacteria culture for MF and mono-colonized bees.

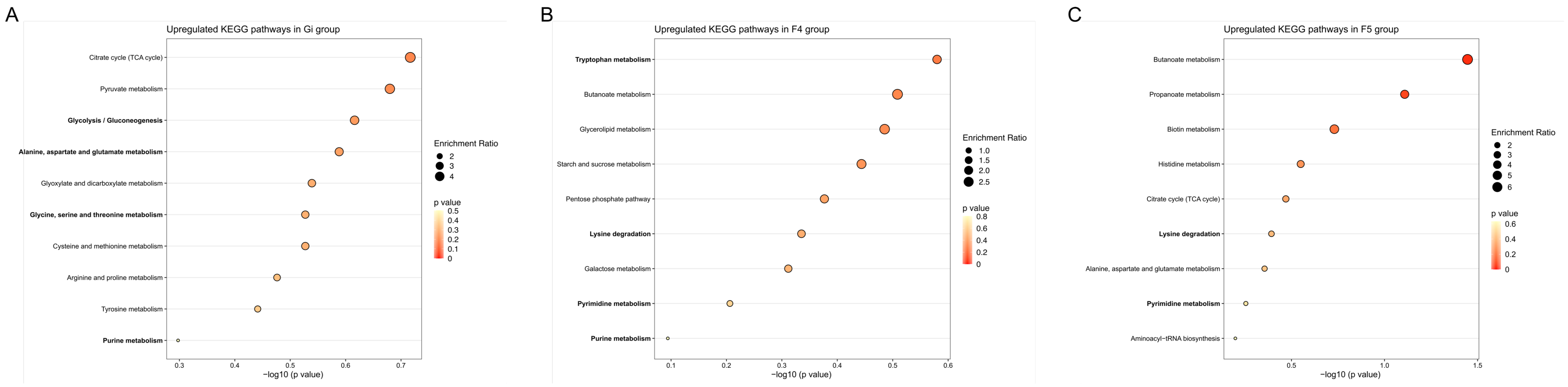

FIG S2 The most significantly enriched KEGG pathways upregulated in the gut of (A) *Gilliamella*, (B) *Lactobacillus* Firm4, and (C) *Lactobacillus* Firm5 inoculated bees

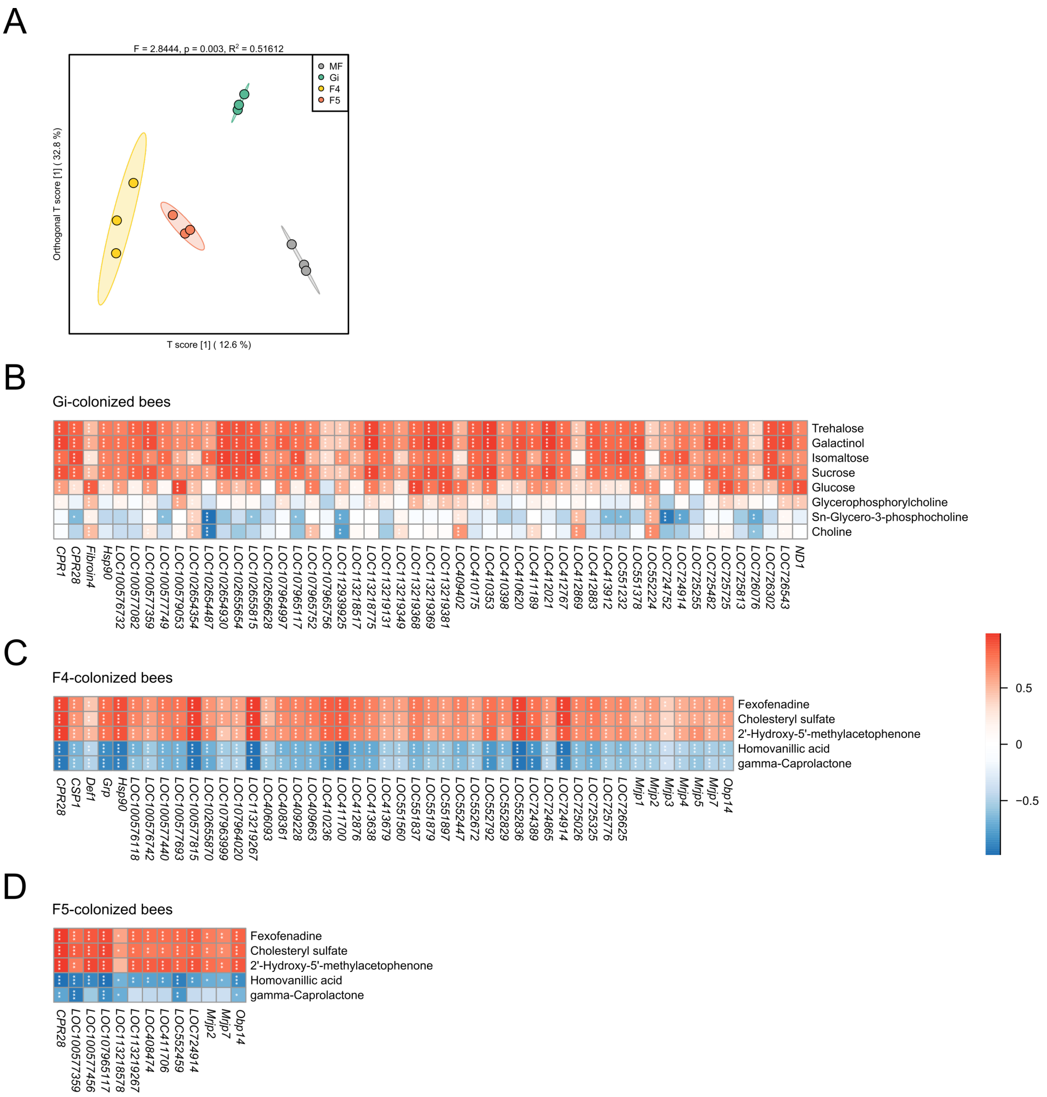

FIG S3 Gut microbiota impacts transcriptomic profile in the honeybee brain. (A) Orthogonal PLS-DA based on normalized gene expression in the brain of microbiota-free and bacteria-colonized bees. Group differences were tested by PERMANOVA. (B-D) Pearson's correlation coefficients between the most related metabolites in hemolymph and upregulated genes in the brain of (B) Gi group, (C) F4 group, and (D) F5 group. The color of red or blue indicates the positive or negative correlation. * $p < 0.05$, ** $p < 0.01$, *** $p < 0.001$.

A

Event types	Gi		F4		F5	
	Total events	Significant events	Total events	Significant events	Total events	Significant events
A3SS	2,206	356	2,264	341	2,274	292
A5SS	1,766	319	1,817	290	1,805	236
MXE	1,544	385	1,610	209	1,590	198
RI	853	165	883	153	873	146
SE	9,244	1,250	9,640	1,138	9,512	923

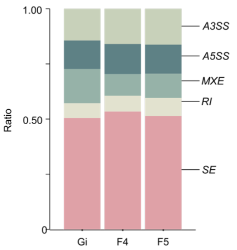

B

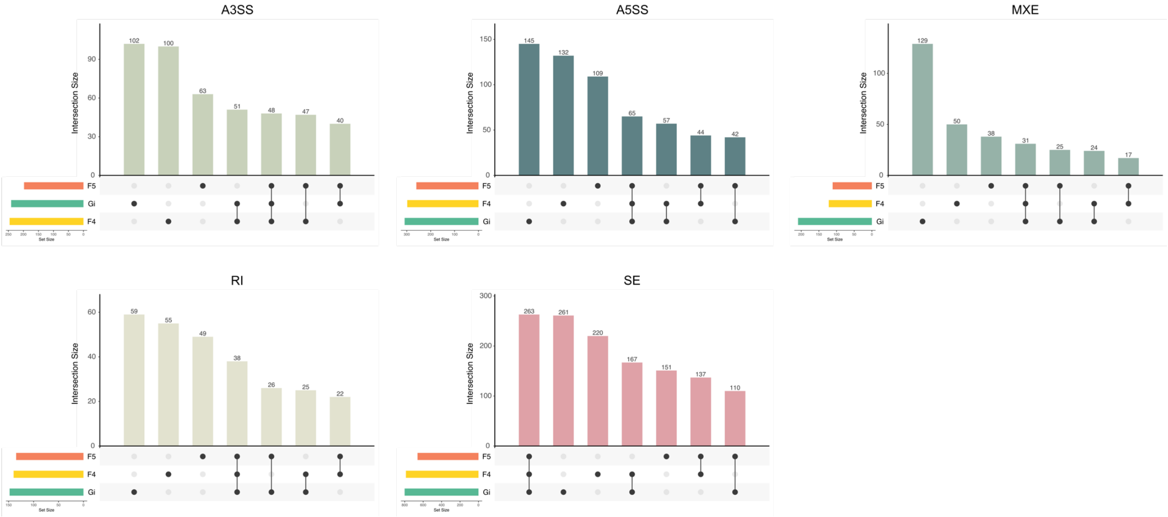

FIG S4 Gut microbiota impacts spliced genes in the honeybee brain. (A) Numbers of the differential alternative splicing events in the brains of bacteria-colonized bees compared to MF bees. Stacked column graph shows the relative abundance of different types of alternative splicing events in each group. A3SS, alternative 3’ splice site; A5SS, alternative 5’ splice site; MXE, mutually exclusive exon; RI, retained introns; SE, skipped exon. (B) UpSet plots showing the intersections of alternative splicing (AS) events associated with different bacteria-colonized groups. The dots and lines on the bottom right represent which intersection is shown by the bar plots above. The size of intersections is given above the bar plot. The total amount of different types of events for each bee group is given to the left of the intersection diagram.

Table S1 (separate file). Raw data of all metabolites abundance in the hemolymph of microbiota-free and bacteria-colonized bees.

Table S2 (separate file). Hemolymph metabolomic WGCNA module analysis of microbiota-free and bacteria-colonized bees.

Table S3 (separate file). Normalized gene expression levels in brains of microbiota-free and bacteria-colonized bees.

Table S4 (separate file). Alternative splicing events in brains of microbiota-free and bacteria-colonized bees.
